# Are parents really attached to their adopted children?

**DOI:** 10.1186/2193-1801-3-545

**Published:** 2014-09-22

**Authors:** Muhammad Imtiaz Subhani, Amber Osman, Fariha Abrar, Syed Akif Hasan

**Affiliations:** Office of Research, Innovation & Commercialization- ORIC, Iqra University-IU, Main Campus, Karachi Pakistan; State City University of New York, New York, USA; Iqra University-IU, Main Campus, Karachi, Pakistan

**Keywords:** Attachment theory, Adopted children, Secure attachment behavior, Avoidant attachment behavior, Ambivalent attachment behavior, Disorganized attachment behavior

## Abstract

**Background:**

Psychological studies found that adopted children suffer from lack of attachment relationships in life. It is important for new parents to understand the underlying concepts before they begin to comprehend behavior issues arising out of different turbulent situations in an adopted child’s life. Attachment theory facilitates in comprehending the frame of mind of these children, when they come from emotionally turbulent backgrounds and how some, if not all behavior issues can be attempted to be resolved to recognize children better and to create a nurturing relationship between adopted child and new parents.

**Findings:**

Focus group method was deployed to collect the data via un-restricted non-probability sampling approach; data was quantified for evaluating the hypotheses via t-test of equality of means. Cross cultural findings suggested that parents-adopted children relationship in terms of secure attachment is revealed more in non-working parents, female parents, children of 11-14 years and female children across stated nations while, the ambivalent, avoidant and disorganized attachments are found more in practice if parents are working & male parents and if foster children are male at large & of 15-18 years.

**Conclusion:**

It is concluded that the task of creating an enriched attachment relationship with an adopted child depends more on parents, normally non working parents and female parents while quality time and care is given somehow the other to young and female kids by either of the parents for establishing quality attachment. Quality time being bestowed to kids translates the category and intensity of parents- children associations.

## Introduction: a view of attachment theory

The term attachment or attachment relationship means in order to create one with a child which is being adopted. Adopted children in most cases go through trauma, separation due to loss of a parent or parents (death/divorce/financial burden) and then are often placed for adoption which affects their attachment relationships with new parents or caregivers (Frances 1965). The children in orphan homes/foster homes are described by Levy (1937) as apparently charming and affectionate. Provence and Lipton (1962) explained foster children as promiscuously friendly. The attachments theory by Bowlby (1958); Harlow and Zimmermann (1958) suggested that children are pre-programmed since birth to form attachments with others as this will help them to live on. The behaviors of the little ones (infants), which are known as ‘social releaser’ such as smiling and crying that rouse inherent care giving reactions from grown-ups (adults). The determining factor of attachment is not food but concern and responsiveness.

Attachment theory developed by (BowlbyI980a) explained early childhood development and lays tremendous importance that a human infant has a biological need for protective attachment figure for survival and absence of such a figure can cause psychological difficulties in the child’s emotional growth. His researches were mostly observations of infants up to formative age (6 years) in a child’s life. To understand and study the different types of attachments children may have with their parents, Ainsworth and Wittig (1969) developed an assessment technique known as ‘Strange Situation Classification’. This technique was earlier studied by Ainsworth (1967) for Uganda studies and Baltimore studies (Ainsworth et al. 1971).

(Bowlby 1973) was further expanded by Ainsworth (1978) when she worked with him and came up with the Strange Situation Test in which she observed infants as young as 8 months old in a controlled environment along with a parent such as mother in that room. During the test they briefly introduced a stranger in the room while the mother left for a few minutes and then she returned. They were able to conclude from this test that infants are capable of different reactions when their mother leaves and joins them again or when a stranger is introduced temporarily instead of a mother. They recorded reactions such as crying, screaming, going to the door when mother leaves them and on her return they are calm and back to exploring their environment. Some of them were also not as responsive to missing their mother and reacted aloof on their mother’s return.

### Attachment behaviors

There are few types of attachment behaviors. Attachment theory describes them as:

1. Secure
2. Avoidant
3. Ambivalent
4. Disorganized

A *secure attachment* with a parent or caregiver is considered to be the most positive one where the caregiver is responsive to the child completely and makes the child feel secure. Bowlby (1988) further explained that such positive attachment experience let the child form future relationships influenced by early childhood experiences. It endorses the feeling of approval by their caregiver making them feel good about themselves ( for e.g. ‘I am loved’) (Oppenheim and Goldsmith 2007).

In *avoidant attachment* children try to be caretakers in the relationship as their parents are not responsive to their needs. They have parents who frighten the child and are the source of comfort as well as fear which develops another set of behavior pattern where the child is unable to formulate his behavior and thinking.

In *ambivalent attachment* children have a difficult time dealing with their anger and resentment towards their parent. Their parents don’t connect to them emotionally and are inconsistent, giving rise to anxiety and frustration in a child’s behavior.

In *disorganized attachment* children experienced no positive early childhood relationships due to neglect, lack of presence of attachment figures or abuse (Bowlby et al. 1956).

Attachment behaviors are necessary in order to create some meaning out of the world around the child, to develop certain emotional attachment in their relationships such as:

1. It helps child to understand how to relate to others.
2. It gives a sense of feelings towards one’s own self.

For the strange situation classification, (Ainsworth et al. 1971) concluded that the behavior was assessed on the basis of the behavior of the primary care taker, which was mother in this matter. For instance, emotionally involved infant are related with receptive & sensitive primary care.

Incompatible primary care is found with insecure ambivalent attached infants. In this case, sometimes the child’s wants, desires etc are met but sometimes they are overlooked by the father/mother.

Insecure avoidant infants are related to more insensitive primary case. The child believes that his/her wants, desires etc has no persuasion on the father/mother.

(Ainsworth and Bell 1970) conclusion presented the first experimental proof for Bowlby’s (1969) attachment theory. For example, securely attachment children have an optimistic working model and mental demonstration of others as being supportive while also considering themselves respectful (Jacobsen and Hoffman 1997).

The avoidant children consider themselves as an undeserving and unacceptable due to primary caregivers’ snubbing behavior (Larose and Bernier 2001).

Ambivalent children have pessimistic self image and overstate their poignant responses as means to gain attention (Kobak et al. 1993). Insecure attachment style in related with increased emotional and social behavioral troubles.

The strange situation test is taken as a reliable test to assess the attachments of children. For example, there was a study conducted in Germany in which 78% children were categorized in the same way at ages 1 till 6 years (Wartner et al. 1994).

According to Melhuish (1993), the strange situation is a method widely used to identify the infant attachment to a caregiver. Lamb (1977) pointed out as being artificial and lacks environmental validity. The reason is that the child is placed in an artificial environment and the process of mother entering and leaving room follows a preset script. Lamb (1977) criticized that Ainsworth strange situation experiment only studies the child behavior with mother and not the other types of attachments to a father, grandmother etc. Marrone (1998) explained that though the Strange Situation has been pointed out for being stressful for children but it is also modeling daily experiences, as mothers do go away from their babies for a short span of time in different settings and often also leave them with new people for instance baby sitters.

### Understanding what is adoption

Adoption is a process by which an individual can adopt a child becoming primary caregiver and is able to assume all rights and responsibilities from the original parents. Children are placed for adoption sometimes by their own parents, relatives or different agencies depending on the circumstances (Bartholet 1993). Sometimes it is death of a parent/primary caregiver, other times financial constraints makes parents take the decision of putting their child for adoption in the best interest of the child or some children are neglected and abused by their parents forcing them to be separated from them. Adoption is seen as the most favorable option for children who have been neglected, abused or suffering from trauma (Oppenheim and Goldsmith 2007).

A flourishing adoption is that when the child in time or steadily develops a secure attachment with his/her new family or care giver. Most children are able to develop the bonds and as a result, this attachment becomes the basis for mixing into family and for their psychosomatic development (Hughes 1999). Sometimes, children face problems while settling down in new homes, having various placements, which brings in gaps in their development to form attachments with their care-givers, irrespective of how caring and loving their care-givers or families are. It is important that these children are aided in a specialized manner and programming so that they can become part of families (Hughes 1999).

Findings which used story-stem procedures (Hodges et al. 2003) concluded that children who experienced violence, death, bloodshed had their thinking set in a disturbing pattern at the time of being placed for adoption and had no faith that any adult can be trusted around.

Disturbance in adoption does not mean that only the child has attachment issues but can be many other issues. For e.g. the parents who are adopting a child is not good at parenting or there is a mismatch between the family and the child. Also, the child has been given for adoption at different places for adoption and that is creating a disrupting behavior translating into anxiety about forming an attachment (Hughes 1999).

It is important to have good skill set and highly committed people working in the adoption agencies as they are responsible for placing a child in a new home. The same child experiences positive or negative attachment behaviors. These agencies should also cater to the proper rules and procedures for minimizing the attachment problems children have in their new homes; agencies should make the new parents fully aware about the child attachment issues, help the child needs and might need in future. Also, parents level of attention required by the child so that he/she is able to form an attachment. It has been seen that ignorance and mistreatment towards the foster children through their past experiences of bad homes, real parents mal-treatment etc. has brought them to an extreme traumatic illness, which causes gaps in their development of positive secure attachment by their new parents (Hughes 1999).

### Difficulties in developing attachment with adopted children

Children placed in adoption sometimes have experienced insecure early relationships from their biological families and often have not received proper parenting due to which they often have difficulty with emotional regulation and they might not be able to develop empathy, social understanding or moral development as they were never exposed to make sense of these emotions. They might have developed poor cognitive skills, and have not cultivated the ability of learning from others.

Developing a new attachment with a child who faced neglect by their attachment figures is challenging. Adopted child must have an older memory built in from the previous relationship which must be insecure and they must have learned to distort or adapt their behavior in order to seek attention or suppress their feelings in front of new parents. Before adoption in some cases children are frightened, neglected or abused by their attachment figures. In other instances children might become aggressive and don’t know how to control their anger, resentment or feeling being ‘given up ‘for adoption, feeling unwanted.

The new caregivers then would need to grasp the concept underlying to connect to the adopted child emotionally. This interaction stimulates brain development in regards to relationships and it starts to develop and organize certain context which builds further modulation of emotion and capacity of forming relationships (Testa 2004).

If the child does not develops theory of mind then it blocks the ability of the child to create a new bond with the new caregivers in his life after being adopted. They might not associate with the new parent and not participate fully as a member of their new family.

“Orphans adopted from Romania were adopted in extremely supportive homes and many di d well, there still was a limit to the amount of change that was possible” (Rutter et al. 2007, p. 181; Zeanah et al. 2005).

### Nurturing new attachment relationship with adopted children

To help nurture an attachment relationship, new parents have to create family setting which shows that they can be trustworthy, sensitive parents. Certain tools can help to achieve closeness.

They have to show *empathy* to the child; try to develop regulation by helping him with his emotions and giving opportunity of developing new experiences in relationships. They have to encourage the child to develop a *sense of belonging* in the family by making them participate in family activities. Certain routines such as dinner together at the family table or a family picnic could help them feel part of the family (Sears and Sears 2001).

Parents can designate them their own place and items such as their own room, own toys which makes them have a *claim* in the family with other members and gives them a comfort of knowing that they are here permanently. It is also important to keep a *calm atmosphere* at home where the adopted child is given clear guidelines of what expectations the parents have and he understands the boundaries and parents should avoid confrontations in front of other members if the child misbehaves. This provides consistency in the upbringing (Festinger 2002).

### Description of data, sample and hypotheses

The sampling plan of this study was to investigate the parents and adopted children relationship in terms of attachment behaviors the parents endure while adopting a child from fosterage community. Survey is used to collect the relevant data from large sample of the population (McIntyre 1999, p. 74). The data was collected through focus groups from the respondents (adopted children) of two Eastern (Pakistan and China) and two western countries (United States and United Kingdom). The survey was designed for parents and adopted children relationships in terms of four constructs of attachments: Secure, Avoidant, Ambivalent and Disorganized attachments. The method used included 60 focus groups having 8 adopted children (ages 11-18 yrs.) in each group, having the sample size of 1920 respondents. Most of the focus groups had equal number of males and females adopted children, (1 focus group = 4 male children and 4 female children) but 18 focus groups had random number of males and females adopted children for e.g. 6:2 male: female; 3:5 male: female. The male parents of all the adopted children were all working parents and the female parents of all the adopted children were working and non-working parents both. All the children were randomly picked for this research. These children have spent certain amount of time in the orphanage houses/foster homes but we didn’t focus on the duration of the time spent by these children with the foster homes as that was not included in the focus of our research but it was evident by talking to these children that they were aware that they do have their real parents and their new parents are adopting them because they wish to have a family which is only complete when husband and wife becomes parents of child.

The questions were designed to ask the types of attachments in which they are into with their caregivers. Un-restricted non probability sampling method was used in relevance to the outlined constructs. The questions were made according to the Iqra University’s research committee’s approval. The subject of this research was about one’s belief, attitudes and behaviors of adopted children as discussed in the above section. It is always difficult to comprehend subjective responses and opinions hence, to recognize the adequacy and coherency of the answers, sequence of connected questions were designed (Morgan, 2010). The data which were collected from the stated respondents were with several items and nonparametric in nature while they have been converted into parametric by averaging them out.

Independent sample t-test (t-test for equality of means) was used after splitting the data country wise, to evaluate the hypotheses of this study which were formulated after finding the gap in the existing literature, which include:

H1: The parents and adopted children relationship in terms of secure attachment is more in non-working parents than the working parents.

H2: The parents and adopted children relationship in terms of avoidant attachment is more in non-working parents than the working parents.

H3: The parents and adopted children relationship in terms of ambivalent attachment is more in non-working parents than the working parents.

H4: The parents and adopted children relationship in terms of disorganized attachment is more in non-working parents than the working parents.

H5: The parents and adopted children relationship in terms of secure attachment is more in female parents than the male parents.

H6: The parents and adopted children relationship in terms of avoidant attachment is more in female parents than the male parents.

H7: The parents and adopted children relationship in terms of ambivalent attachment is more in female parents than the male parents.

H8: The parents and adopted children relationship in terms of disorganized attachment is more in female parents than the male parents.

H9: The parents and adopted children relationship in terms of secure attachment is more in younger children than the elder children.

H10: The parents and adopted children relationship in terms of avoidant attachment is more in younger children than the elder children.

H11: The parents and adopted children relationship in terms of ambivalent attachment is more in younger children than the elder children.

H12: The parents and adopted children relationship in terms of disorganized attachment is more in younger children than the elder children.

H13: The parents and adopted children relationship in terms of secure attachment is more in female children than the male children.

H14: The parents and adopted children relationship in terms of avoidant attachment is more in female children than the male children.

H15: The parents and adopted children relationship in terms of ambivalent attachment is more in female children than the male children.

H16: The parents and adopted children relationship in terms of disorganized attachment is more female children than the male children.

### Empirical findings

The findings of this study are explained via Tables 1, 2, 3 and 4, while Table 5 explains the assessment of all hypotheses through extracted findings. Table 1 specifically reports the parents and adopted children relationship in terms of secure, avoidant, ambivalent and disorganized attachments for non working and working parents country-wise and suggests that the secure attachment is more practicing attachment in the parents and adopted children relationship for non-working parents than the working ones across the selected cultures as the mean values of secure attachment is found more for nonworking parents than the working parents at p-value < 0.05 for all outlined selected nations which includes Pakistan, China, US and UK. Thus, the Hypothesis 1 (H1) got accepted for all selected nations as stated in Table 5 (i.e. hypotheses assessment summary).

**Table 1:** **Country**-**wise means for various attachments of parents with adopted children in terms of parents**’ **working**/**nonworking status**

Types of attachments	Status of parents	Pakistan	China	United States	United Kingdom
*Secure attachment*	Non-Working Parents	0.31	0.20	0.13	0.17
	Working Parents	0.15	0.11	0.07	0.06
*Avoidant attachment*	Non-Working Parents	0.05	0.05	0.08	0.09
	Working Parents	0.30	0.21	0.20	0.15
*Ambivalent attachment*	Non-Working Parents	0.08	0.06	0.10	0.11
	Working Parents	0.21	0.19	0.24	0.20
*Disorganized attachment*	Non-Working Parents	0.09	0.03	0.05	0.04
	Working Parents	0.11	0.15	0.13	0.18

**Table 2:** **Country**-**wise means for various attachments of parents with adopted children in terms of parents**’ **gender**

Types of attachments	Gender of parents	Pakistan	China	United States	United Kingdom
*Secure attachment*	Female	0.21	0.14	0.10	0.15
	Male	0.12	0.06	0.09	0.05
*Avoidant attachment*	Female	0.13	0.07	0.04	0.12
	Male	0.14	0.18	0.23	0.14
*Ambivalent attachment*	Female	0.15	0.08	0.11	0.12
	Male	0.17	0.16	0.20	0.16
*Disorganized attachment*	Female	0.02	0.17	0.11	0.08
	Male	0.06	0.14	0.12	0.19

**Table 3:** **Country**-**wise means for various attachments of parents with adopted children in terms of age wise status of children**

Types of attachments	Status of children in terms of age	Pakistan	China	United States	United Kingdom
*Secure attachment*	11-14	0.21	0.19	0.10	0.09
	15-18	0.16	0.14	0.01	0.06
*Avoidant attachment*	11-14	0.10	0.10	0.15	0.11
	15-18	0.12	0.17	0.18	0.17
*Ambivalent attachment*	11-14	0.11	0.09	0.14	0.10
	15-18	0.13	0.14	0.13	0.18
*Disorganized attachment*	11-14	0.04	0.06	0.13	0.11
	15-18	0.05	0.11	0.15	0.18

**Table 4:** **Country**-**wise means for various attachments of parents with adopted children in terms of gender of children**

Types of attachments	Gender of children	Pakistan	China	United States	United Kingdom
*Secure attachment*	Female	0.22	0.17	0.06	0.09
	Male	0.15	0.12	0.03	0.05
*Avoidant attachment*	Female	0.09	0.11	0.19	0.14
	Male	0.14	0.19	0.21	0.16
*Ambivalent attachment*	Female	0.10	0.07	0.14	0.13
	Male	0.12	0.13	0.16	0.15
*Disorganized attachment*	Female	0.11	0.09	0.11	0.13
	Male	0.07	0.12	0.10	0.15

**Table 5:** **Hypotheses assessment summary**

Hypotheses	Pakistan	China	United States	United Kingdom
	Comparisons for Means for Non working parents VS Working parents with (2-tailed significance/ p-value) & empirical conclusion			
H1: The parents and adopted children relationship in terms of secure attachment is more in non-working parents than the working parents.	0.31 > 0.15 (0.000) Accepted	0.20 > 0.11 (0.000) Accepted	0.13 > 0.07 (0.000) Accepted	0.17 > 0.06 (0.000) Accepted
H2: The parents and adopted children relationship in terms of avoidant attachment is more in non-working parents than the working parents.	0.05 < 0.30 (0.000) Rejected	0.05 < 0.21 (0.000) Rejected	0.08 < 0.20 (0.000) Rejected	0.09 < 0.15 (0.000) Rejected
H3: The parents and adopted children relationship in terms of ambivalent attachment is more in non-working parents than the working parents.	0.08 < 0.21 (0.000) Rejected	0.06 < 0.19 (0.000) Rejected	0.10 < 0.24 (0.000) Rejected	0.11 < 0.20 (0.000) Rejected
H4: The parents and adopted children relationship in terms of disorganized attachment is more in non-working parents than the working parents.	0.09 < 0.11 (0.000) Rejected	0.03 < 0.15 (0.000) Rejected	0.05 < 0.13 (0.000) Rejected	0.04 < 0.18 (0.000) Rejected
	Comparisons for Means for Female parents VS Male parents with (2-tailed significance/ p-value) & empirical conclusion			
H5: The parents and adopted children relationship in terms of secure attachment is more in female parents than the male parents.	0.21 > 0.12 (0.000) Accepted	0.14 > 0.06 (0.000) Accepted	0.10 > 0.09 (0.000) Accepted	0.15 > 0.05 (0.000) Accepted
H6: The parents and adopted children relationship in terms of avoidant attachment is more in female parents than the male parents.	0.13 < 0.14 (0.079) Rejected	0.07 < 0.18 (0.000) Rejected	0.04 < 0.23 (0.000) Rejected	0.12 < 0.14 (0.056) Rejected
H7: The parents and adopted children relationship in terms of ambivalent attachment is more in female parents than the male parents.	0.15 < 0.17 (0.103) Rejected	0.08 < 0.16 (0.000) Rejected	0.11 < 0.20 (0.000) Rejected	0.12 < 0.16 (0.049) Rejected
H8: The parents and adopted children relationship in terms of disorganized attachment is more in female parents than the male parents.	0.02 < 0.06 (0.000) Rejected	0.17 > 0.14 (0.047) Rejected	0.11 < 0.12 (0.105) Rejected	0.08 < 0.19 (0.000) Rejected
	Comparisons for Means for Younger children VS Elder children with (2-tailed significance/p-value) & empirical conclusion			
H9: The parents and adopted children relationship in terms of secure attachment is more in younger children than the elder children.	0.21 > 0.16 (0.000) Accepted	0.19 > 0.14 (0.000) Accepted	0.10 > 0.01 (0.000) Accepted	0.09 < 0.06 (0.000) Accepted
H10: The parents and adopted children relationship in terms of avoidant attachment is more in younger children than the elder children.	0.10 < 0.12 (0.061) Rejected	0.10 < 0.17 (0.000) Rejected	0.15 < 0.18 (0.030) Rejected	0.11 < 0.17 (0.000) Rejected
H11: The parents and adopted children relationship in terms of ambivalent attachment is more in younger children than the elder children.	0.11 < 0.13 (0.070) Rejected	0.09 < 0.14 (0.000) Rejected	0.14 > 0.13 (0.155) Rejected	0.10 < 0.18 (0.000) Rejected
H12: The parents and adopted children relationship in terms of disorganized attachment is younger children than the elder children.	0.04 < 0.05 (0.180) Rejected	0.06 < 0.11 (0.000) Rejected	0.13 < 0.15 (0.101) Rejected	0.11 < 0.18 (0.000) Rejected
	Comparisons for Means for Female children VS Male children. with (2-tailed significance/ p-value) & empirical conclusion			
H13: The parents and adopted children relationship in terms of secure attachment is more in female children than the male children.	0.22 > 0.15 (0.000) Accepted	0.17 > 0.12 (0.000) Accepted	0.06 > 0.03 (0.030) Accepted	0.09 > 0.05 (0.027) Accepted
H14: The parents and adopted children relationship in terms of avoidant attachment is more in female children than the male children.	0.09 < 0.14 (0.170) Rejected	0.11 < 0.19 (0.000) Rejected	0.19 < 0.21 (0.020) Rejected	0.14 < 0.16 (0.003) Rejected
H15: The parents and adopted children relationship in terms of ambivalent attachment is more in female children than the male children.	0.10 < 0.12 (0.160) Rejected	0.07 < 0.13 (0.000) Rejected	0.14 < 0.16 (0.001) Rejected	0.13 < 0.15 (0.010) Rejected
H16: The parents and adopted children relationship in terms of disorganized attachment is more female children than the male children.	0.11 > 0.07 (0.002) Accepted	0.09 < 0.12 (0.050) Rejected	0.11 > 0.10 (0.090) Accepted	0.13 < 0.15 (0.090) Rejected

Table 1 further explains that the parents and adopted children relationship in terms of avoidant, ambivalent and disorganized attachments are found to be more in the working parents than the non working parents for all stated nations as the means values of these attachments for working parents are significantly huskier than the non working parents, thus we fail to accept the Hypotheses 2, 3 and 4 as also explained in Table 5 for all selected cultures.

Table 2 reports the parents and adopted children relationship in terms of secure, avoidant, ambivalent and disorganized attachments for female and male parents country-wise. It is confirmed in this table the secure attachment, is again a more practicing attachment in the parents and adopted children relationship in female parents than the male parents across the selected nations as the mean values of secure attachment is found more for female parents than the male parents at p-value < 0.05 for all outlined selected nations thus, the Hypothesis 5 (H5) got accepted for all selected nations as mentioned in Table 5. Whereas, avoidant, ambivalent and disorganized attachments are found more in the male parents than the female ones for all stated nations as the means values of these attachments for male parents are significantly larger than the female parents, thus we fail to accept the Hypotheses 6, 7 and 8 as also explained in Table 5.

Table 3, highlights the parents and adopted children relationship in terms of secure, avoidant, ambivalent and disorganized attachments for younger and elder adopted children country-wise. Table 3 reports that the secure attachment always is a more practicing attachment in the parents and adopted children relationship in younger children than the elder ones across the selected nations as the mean values of secure attachment is found more for younger than the elder children at p-value < 0.05 for all outlined selected nations thus, we fail to reject the Hypothesis 9 (H9) i.e. the parents and adopted children relationship in terms of secure attachment is more in younger children than the elder children, for all selected nations as mentioned in Table 5. The parents and adopted children relationship in terms of avoidant, ambivalent and disorganized attachments are found more in elder children than the younger children, for all selected nations as the means values of these attachments for younger children are not significantly larger than the elder children, thus we fail to accept the Hypotheses 10, 11 and 12 as also mentioned in Table 5.

The Table 4 highlights the parents and adopted children relationship in terms of secure, avoidant, ambivalent and disorganized attachments for female and male kids country-wise. Table 4 confirms that the parents and adopted children relationship in terms of secure attachment is more in female children than the male ones as the mean values of secure attachment for this category is found more for female children than the male kids at p-value < 0.05 for all outlined selected nations thus, we fail to reject the Hypothesis 13 (H13) i.e. the parents and adopted children relationship in terms of secure attachment is more in female children than the male children, for all selected nations as mentioned in Table 5. While, the avoidant and ambivalent attachments are found more in male children than the female children, empirically at p < 0.05, for China, US and UK and at p < 0.2 for Pakistan therefore, we fail to accept the hypotheses 14 and 15 at the stated testing specifications for all selected cultures as mentioned in hypotheses assessment summary (Table 5). It should be noted that the parents and adopted children relationship in terms of disorganized behavior are found empirically more in female kids than the male ones for Pakistan at p < 0.05 and for US at p < 0.10 whereas, empirically more in male kids than the female ones for China and UK at p < 0.10. Thus, we fail to reject the hypothesis 16 (H16) for Pakistan and US and fail to accept it for China and UK at the stated specifications as stated in hypotheses assessment summary/Table 5.

## Conclusion

The task of creating an enriched attachment relationship with an adopted child depends more on the parents, as shown in the findings of this paper that normally, non working parents and female parents they are enabled to give a quality time to the kids after adopting them while quality time and care is given somehow the other to young kids and female kids by either of the parents for establishing quality attachment across the selected cultures. The quality time bestow to kids translates the category and intensity of parents- children associations. Creating an attachment relationship with a child who is adopted as an infant is relatively uncomplicated compared to a child who has been exposed to various circumstances earlier such as neglect, trauma or loss of parent. They might have not had any secure relationships with any attachment figures in early life or have already developed within different relationships, which makes them feel lost and they are unable to communicate (Freivalds, 2002). They could have their own preconceived notions of their new environment. This makes them withdraw completely from their new family or display aggressive behavior out of resentment which is challenging for the new parents. Some children adapt easily to their new parents and families meanwhile others might have difficulty in coming to terms and expressing themselves. Attachment theory helps in defining some aspects of early childhood behavior which can give an insight to the child’s mind and why they react differently. Change in approach by the parents to the different behavior of the adopted child, could encourage positive attachment behavior from the child. It can help parents to establish the connection which the child has been missing and make the relationship nurture ahead.
